# Perceived Social Support and Cognitive Functions Among First‐Generation Older Asian Americans

**DOI:** 10.1002/alz70860_107042

**Published:** 2025-12-23

**Authors:** Yirou Jiang, Annie Ching, Oanh L. Meyer, Josh D Grill, Bora Nam, Marian Tzuang, Linh Nguyen, Seoyeon Yum, Anson Wong, Sarah Tomaszewski Farias, Boon Lead Tee, Quyen Q Tiet, Haeok Lee, Van Ta Park

**Affiliations:** ^1^ University of California San Francisco School of Nursing, San Francisco, CA, USA; ^2^ University of California, Davis School of Medicine, Sacramento, CA, USA; ^3^ University of California, Irvine, Irvine, CA, USA; ^4^ Memory and Aging Center, Department of Neurology, University of California San Francisco, San Francisco, CA, USA; ^5^ California School of Professional Psychology at Alliant International University, San Francisco, CA, USA; ^6^ Meyers College of Nursing, New York University, New York, NY, USA

## Abstract

**Background:**

Studies suggest perceived social support from family and friends acts as a protective factor against cognitive decline, especially in older adults. However, few studies have focused on first‐generation immigrants in the United States. This study investigates how the perceived social support is differently associated with specific cognitive domains in first‐generation older Asian Americans.

**Method:**

Data were collected from 141 first‐generation older Asian Americans (Chinese, Korean, and Vietnamese) participating in an ongoing ARISE (Asian Americans & Racism: Individual and Structural Experiences) pilot study who are 65 years old or older in California. Participants completed cognitive and psychosocial assessments in their preferred languages (Mandarin, Korean, and Vietnamese). Verbal encoding and memory were assessed using the Auditory Verbal Learning Test. Basic attention and auditory working memory were measured using Digit Span Forward and Backward, respectively. Perceived social support from family and friends was assessed using the 6‐item National Latino and Asian American Study Social Network Scale on a 5‐point Likert scale (1 = strongly disagree, 5 = strongly agree). Multivariable linear regression and moderation analyses were performed, controlling for age, gender, and education.

**Result:**

Participants were aged 65–88 years (Mean = 74.55, SD = 5.40); 50.3% were female, 74.3% had at least a high school education, and 82.8% expressed positive support level. Greater perceived social support was associated with better verbal encoding (*β* = 0.233, *SE* = 0.154, *p* = 0.003), but worse auditory working memory (*β* = ‐0.249, *SE* = 0.039, *p* = 0.001). Depression moderated the relationship between social support and working memory (*β* = ‐0.026, *SE* = 0.012, *p* = 0.033), with the negative impact being more pronounced at higher levels of depression. No significant associations were found for memory or basic attention (*p* = 0.065, *p* = 0.790).

**Conclusion:**

These findings highlight the complex dynamics of social support in cognitive function among older first‐generation Asian Americans. Social support positively influenced verbal encoding, underscoring the importance of support systems for this population. However, more studies are needed to examine the role of mental health conditions, such as depression, in the relationships between social support and working memory.

